# Gain-of-Function Alleles in *Caenorhabditis elegans* Nuclear Hormone Receptor *nhr-49* Are Functionally Distinct

**DOI:** 10.1371/journal.pone.0162708

**Published:** 2016-09-12

**Authors:** Kayoung Lee, Grace Ying Shyen Goh, Marcus Andrew Wong, Tara Leah Klassen, Stefan Taubert

**Affiliations:** 1 Graduate Program in Medical Genetics, University of British Columbia, Vancouver, BC, Canada; 2 Centre for Molecular Medicine and Therapeutics and Child & Family Research Institute, University of British Columbia, Vancouver, BC, Canada; 3 Graduate Program in Cell and Developmental Biology, University of British Columbia, Vancouver, BC, Canada; 4 Faculty of Pharmaceutical Sciences, University of British Columbia, Vancouver, BC, Canada; 5 Department of Medical Genetics, University of British Columbia, Vancouver, BC, Canada; INSERM U869, FRANCE

## Abstract

Nuclear hormone receptors (NHRs) are transcription factors that regulate numerous physiological and developmental processes and represent important drug targets. NHR-49, an ortholog of Hepatocyte Nuclear Factor 4 (HNF4), has emerged as a key regulator of lipid metabolism and life span in the nematode worm *Caenorhabditis elegans*. However, many aspects of NHR-49 function remain poorly understood, including whether and how it regulates individual sets of target genes and whether its activity is modulated by a ligand. A recent study identified three gain-of-function (gof) missense mutations in *nhr-49* (*nhr-49(et7)*, *nhr-49(et8)*, and *nhr-49(et13)*, respectively). These substitutions all affect the ligand-binding domain (LBD), which is critical for ligand binding and protein interactions. Thus, these alleles provide an opportunity to test how three specific residues contribute to NHR-49 dependent gene regulation. We used computational and molecular methods to delineate how these mutations alter NHR-49 activity. We find that despite originating from a screen favoring the activation of specific NHR-49 targets, all three gof alleles cause broad upregulation of NHR-49 regulated genes. Interestingly, *nhr-49(et7)* and *nhr-49(et8)* exclusively affect *nhr-49* dependent activation, whereas the *nhr-49(et13)* surprisingly affects both *nhr-49* mediated activation and repression, implicating the affected residue as dually important. We also observed phenotypic non-equivalence of these alleles, as they unexpectedly caused a long, short, and normal life span, respectively. Mechanistically, the gof substitutions altered neither protein interactions with the repressive partner NHR-66 and the coactivator MDT-15 nor the subcellular localization or expression of NHR-49. However, *in silico* structural modeling revealed that NHR-49 likely interacts with small molecule ligands and that the missense mutations might alter ligand binding, providing a possible explanation for increased NHR-49 activity. In sum, our findings indicate that the three *nhr-49* gof alleles are non-equivalent, and highlight the conserved V411 residue affected by *et13* as critical for gene activation and repression alike.

## Introduction

Nuclear hormone receptors (NHRs) are transcription factors that modulate gene expression in response to extrinsic and intrinsic cues, and they are essential regulators of many developmental and physiological processes [1]. Mammalian Hepatocyte Nuclear Factor 4 alpha (HNF4α) is an illustrative example of an NHR with diverse functions. Genetic analyses in mice have shown that HNF4α is required for normal gastrulation via a functional requirement in the visceral endoderm, for proper terminal hepatocyte differentiation, and for normal function of the adult liver, including key roles in lipid, bile acid, and xenobiotic metabolism. In the pancreatic β-cells, HNF4α is required for normal glucose homeostasis and for β-cell mass expansion during pregnancy [2–5]. In line with HNF4α’s important metabolic regulatory roles, loss-of-function mutations in the human *HNF4A* gene have been linked to maturity onset diabetes of the young (MODY) and to type 2 diabetes [3,6,7].

The lipid regulatory role of HNF4 is evolutionarily ancient. *Drosophila melanogaster* encodes a single HNF4 ortholog (dHnf4) that is required for lipid mobilization and fatty acid β-oxidation, and loss of dHNF4 results in starvation sensitivity due an inability to convert stored fat into energy [8]. The evolutionarily more ancient nematode *Caenorhabditis elegans* encodes a massively expanded NHR family with 284 members, including 269 NHRs that appear to have derived from an HNF4α-like ancestor [9,10]. Most of these NHRs remain uncharacterized, but several appear to regulate metabolism. NHR-69 cooperates with Smad-type transcription factors to modulate glucose levels and insulin signaling [11], and NHR-8, -10, -13, -49, -62, -64, -66, -76, and -80 belong to an expanding group of NHRs that regulate lipid metabolism and/or metabolic gene expression [12–22]. Thus, HNF4-like NHRs regulate lipid metabolism in invertebrate and vertebrate organisms alike.

*C*. *elegans* NHR-49 controls multiple aspects of fatty acid metabolism and is linked to various physiological and molecular phenotypes. *nhr-49* is required to express genes involved in fatty acid β-oxidation, especially upon starvation, and also promotes the expression of the two fatty acid desaturases *fat-5* and *fat-7* [14,15]. By regulating these key enzymes, NHR-49 promotes metabolic reprogramming that allows adaptation to starvation and is also essential for the extended life span of various mutant and transgenic *C*. *elegans* strains [18,19,23,24]. *nhr-49* dependent fatty acid desaturation also appears to contribute to low-temperature adaptation by altering membrane lipid composition and thus membrane fluidity [25]. Additionally, *nhr-49* is required to repress several additional lipid metabolic genes such as sphingolipid breakdown enzymes and lipases, and also regulates various non-lipid metabolism genes [13]. Whether and how these NHR-49 regulated genes contribute to the organismal functions and phenotypes of *nhr-49* mutant worms has not yet been elucidated.

NHRs contain two evolutionarily conserved signature domains: an N-terminal zinc-finger DNA binding domain (DBD) that enables interaction with genomic hormone response elements; and a C-terminal ligand binding domain (LBD) that mediates reversible binding to ligands and also enables NHR dimerization and other protein-protein interactions [1]. Ligand binding induces structural changes that enable NHRs to differentially interact with transcriptional coregulators including coactivators and corepressors. In turn, this allows NHRs to implement specific gene programs upon ligand binding or dissociation. Changes in NHR dimer and NHR:coregulator interactions are thus essential to adapt genome expression in response to altered ligand availability [26–28]. For NHR-49, several functional partner NHRs and one coregulator have been identified to date. Specifically, NHR-13 and NHR-80 appear to cooperate with NHR-49 in the activation of fatty acid desaturase genes, whereas NHR-66 is thought to cooperate with NHR-49 to repress sphingolipid breakdown and lipase genes [13,17,19]. No NHR-49 corepressor has yet been identified, but the Mediator subunit MDT-15 physically binds to NHR-49 and the two proteins share numerous downstream targets, including fatty acid desaturase genes and starvation induced β-oxidation genes [29]. Thus, MDT-15 likely serves as a coactivator for NHR-49. The physical interaction between NHR-49 and these interacting proteins is mediated by the LBD [13,29], but has not yet been characterized in greater detail.

Recently, three dominant *nhr-49* gain-of-function (gof) alleles have been identified that overcome the cold-sensitive phenotype of a *paqr-2/adiponectin receptor* mutant [25]. One of these gof alleles promotes the expression of *fat-7*, which resulted in increased levels of monounsaturated fatty acids that were essential for cold resistance [25]. We reasoned that further study might reveal whether these gof mutations selectively activate fatty acid desaturase genes or whether they indiscriminately influence all *nhr-49* regulated genes, i.e. whether they represent potential specificity determinants within NHR-49. Additionally, as all three alleles are point mutations near NHR-49’s LBD [25], we reasoned that they might alter protein-protein interactions with partner NHRs or MDT-15 to e.g. induce *fat-7*. We thus set out to characterize the gene expression, protein interaction, and *in vivo* phenotypes of these alleles.

## Results

### *nhr-49* gof mutations affect variably conserved residues and locate close to predicted functional motifs

Svensk *et al*. described three gain-of-function (gof) mutations in the *nhr-49* gene, *et7(P479L)*, *et8(S432F)*, and *et13(V411E)* (amino acid numbers refer to the largest NHR-49 isoform, NHR-49C) [25]. All three alleles act in dominant fashion to suppress the cold-sensitivity and a morphological phenotype of the *paqr-2/adiponectin receptor* mutant. For *et8(S432F)*, the mode of *paqr-2* suppression has been described: this mutation causes the upregulation of the known NHR-49 regulated gene *fat-7* and a concomitant increase in unsaturated fatty acid levels [25]. However, other consequences of the *nhr-49* gof alleles have not yet been described and their molecular characterization remains incomplete.

**Fig 1:**
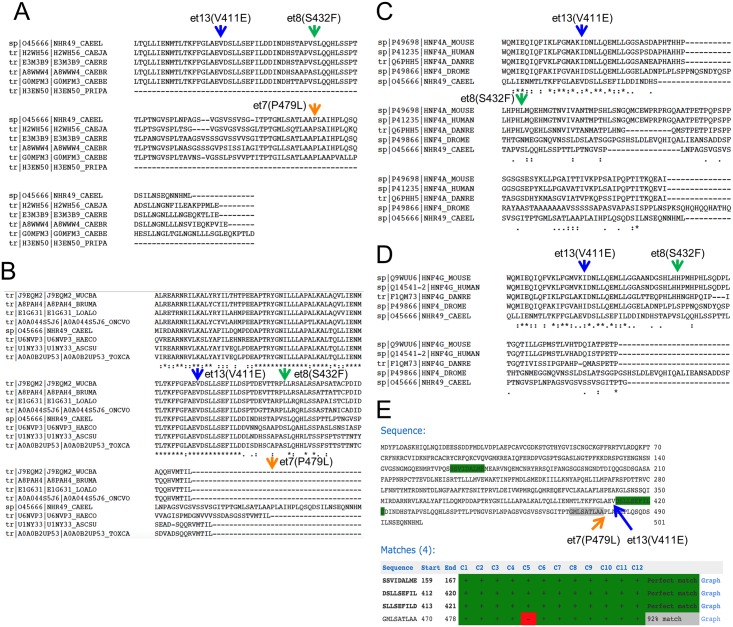
Evolutionary conservation of amino acids affected by *nhr-49* gof alleles. (A-D) CLUSTAL-W alignments of NHR-49/HNF4α/HNF4γ sequences (A) from *C*. *elegans*, *C*. *japonica*, *C*. *remanei*, *C*. *briggsae*, *C*. *brenneri*, and *P*. *pacificus*, (B) *W*. *bancrofti*, *B*. *malayi*, *L*. *loa*, *O*. *volvolus*, *C*. *elegans*, *H*. *contortus*, *A*. *suum*, and *T*. *canis*, and (C, D) *D*. *melanogaster*, *D*. *rerio*, *M*. *musculus*, *H*. *sapiens*, and *C*. *elegans*, with (C) showing alignments to HNF4α and (D) alignments to HNF4γ; residues corresponding to NHR-49C V411, S432, and P479 are indicated by arrows. (E) Predicted 9aaTAD motifs in NHR-49. Note that V411 is adjacent to two overlapping high-confidence 9aaTAD motifs, and P479 is adjacent to an imperfect 9aaTAD motif. The table below the sequence indicates each predicted high stringency 9aaTAD and whether or not it conforms to each of 12 specific refinement criteria of the 9aaTAD prediction algorithm (C1-C12; see [30,31] for details on algorithm and refinement criteria).

To gain better insight into the residues affected by the gof mutations, we aligned the sequence of *C*. *elegans* NHR-49 to the predicted NHR-49 orthologs of other *Caenorhabditis* species (Fig 1A), a group of parasitic nematodes (Fig 1B), and to the HNF4α and HNF4γ orthologs of the arthropod *Drosophila melanogaster*, the vertebrate *Danio rerio*, the mammal *Mus musculus*, and of *H*. *sapiens* (Fig 1C and 1D; see S1 Table for identity/similarity of NHR-49 to *H*. *sapiens* NHRs). As noted previously [25], all three mutations affect residues in or near the ligand-binding domain (LBD) of *C*. *elegans* NHR-49. Our alignments revealed that the three gof alleles arose in residues that exhibit distinct patterns of evolutionary conservation. P479 is identical in the five compared *Caenorhabditis* species, but is missing in all other investigated sequences; S432 is identical in all studied *Caenorhabditis* species and in four of eight queried non-*Caenorhabditis* nematodes (the other four nematodes encode an L at the position), but absent in the higher metazoans analyzed; and V411 is conserved but not identical in all sequences inspected (a V in all tested nematodes, an I in *D*. *melanogaster*, *D*. *rerio*, mouse, and human HNF4). It is therefore likely that the three gof mutations affect distinct properties of the *C*. *elegans* NHR-49 protein; *et13(V411E)* appears particularly interesting as it probably affects an evolutionarily conserved molecular function of NHR-49/HNF4.

### The *et13(V411E)* mutation affects a residue predicted to be involved in the activation/repression switch and in ligand binding

To gain insight into the putative molecular effects of the *nhr-49* point mutations we next inspected whether they are adjacent to predicted functional motifs of NHR-49. Because the gof alleles all lie within or near the LBD, which is involved in transcriptional activation and repression, we assessed whether they are situated near transcriptional activation sequences, specifically the Nine Amino Acids Transactivation Domain (9aaTAD) [30,31]. The 9aaTAD is a structurally and experimentally defined motif that occurs in transcription factors that bind KIX-domain containing coregulators such as p300, CREB binding protein (CBP), and MED15, the ortholog of the NHR-49 coregulator MDT-15 [29,32]. We found that V411 (mutated in *et13(V411E)*) is situated immediately N-terminal to a sequence (DSLLSEFIL) that perfectly matches a 9aaTAD, and that P479 (mutated in *et7(P479L)*) lies immediately C-terminal to a near-perfect (8/9 amino acid) 9aaTAD match (Fig 1E; see Material and Methods for 9aaTAD prediction). Thus, *et7(P479L)* and *et13(V411E)* might influence transcription by altering 9aaTAD function, i.e. interaction with the KIX-domain containing coactivator MDT-15 and/or other coregulators.

**Fig 2:**
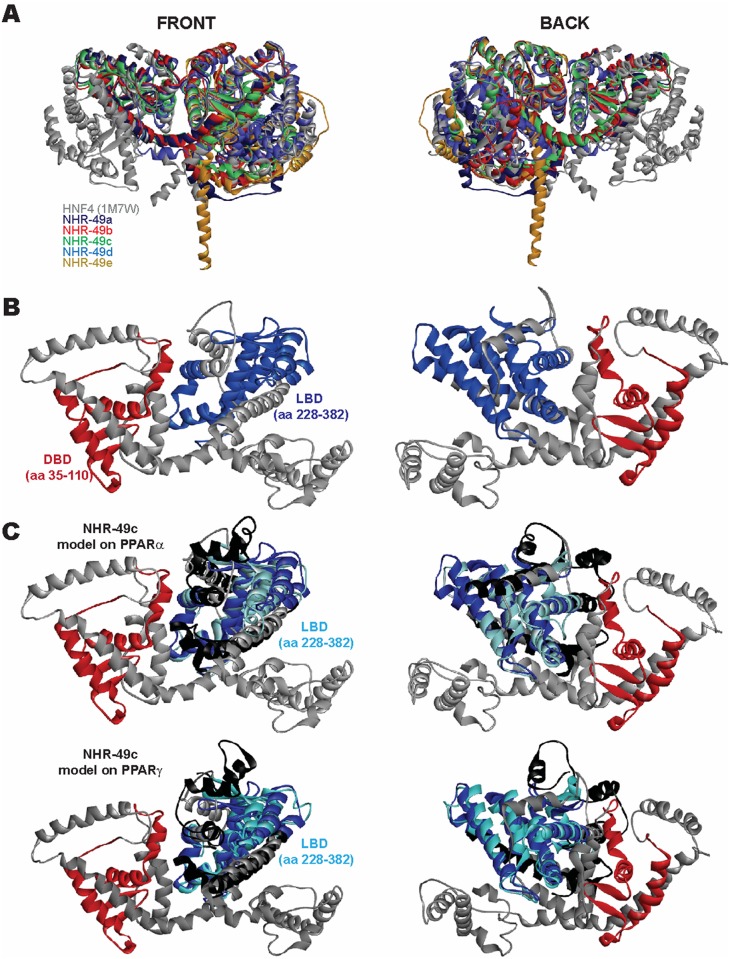
*In silico* modeling of all five NHR-49 isoforms. (A-B) Homology models of the five NHR-49 isoforms generated using the HNF4α crystal structure (PDB 1M7W) depicted in a structural overlay (A). Strong structural similarity is observed for both the DBD (red) and the LBD (blue) for the longest NHR-49C isoform (B), whereas other regions show variability across isoforms. (C) Overlay of the HNF4α-derived NHR-49C model with models generated using the same modeling parameters but experimentally derived PPARα (PDB 2REW; top) and PPARγ (PDB 3E00; bottom) structures as templates (PPAR derived structure: black; LBD: cyan). Both structures successfully model the NHR-49C-LBD (182 aa homology in both models), but neither has the robustness of the HNF4α-derived NHR-49C model. The PPARα-derived model had a 1.6535 RMSD and the PPARγ-derived model a 1.7144 RMSD from HNF4α-derived NHR-49C, indicating a worse overall fit.

As *et13(V411E)* lies within the LBD, an evolutionarily conserved and structurally defined domain, we performed structural homology modeling of NHR-49 to gain further insight into the putative effects of the V411E substitution; we also modeled the effects of an E327A mutation that is predicted to abrogate NHR-49 dimerization [33]. Although no experimentally derived structure is currently available for NHR-49, the structures of HNF4α and HNF4γ have been solved [34–36] and allow *in silico* modeling of NHR-49. As expected, all five NHR-49 isoforms could be modeled onto existing HNF4 structures with high confidence; these models include the DBD and the LBD (Fig 2A and 2B). For all five isoforms (NHR-49A-E) the *Caenorhabditis* specific C-terminal extension of the LBD, which harbors the residues S432 and P479, were also resolved fully. The inherent amino acid sequence differences across isoforms result in distinct quaternary structures in the C-termini of the respective isoforms with similar architectures for the DBD and LBD domains.

NHR-49 shares some functional similarity with mammalian PPARα, and thus it is sometimes considered a functional ortholog of that NHR [14,15,18]. Therefore, we modeled NHR-49C onto existing, experimentally derived PPARα and PPARγ structures (Fig 2C). Although we again obtained high confidence models, these were inferior to the one generated using HNF4α as template, as expected given the higher primary sequence similarity between NHR-49 and HNF4α (S1 Table).

**Fig 3:**
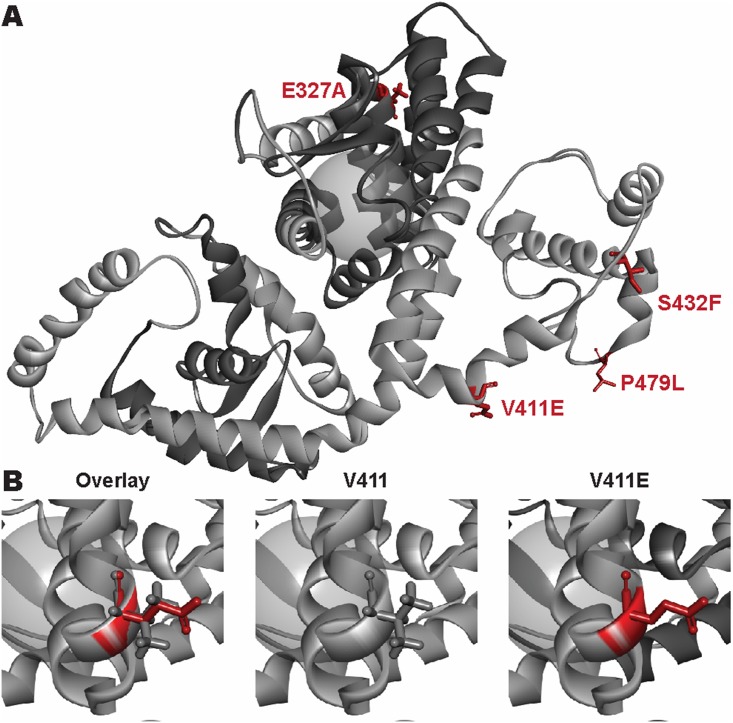
Biophysical effects of gof point mutations in NHR-49. (A) Structural homology model of NHR-49C showing the relative locations of the gof (V411E, P479L, S432F) and predicted lof point mutations (E327A) in red with the DBD and LBD (dark grey) and ligand binding zone (grey sphere). (B) Comparison of the biochemical differences between the wild-type V411 (grey) and mutant E411 (red) amino acid residue. The gof mutation has a substantially longer, negatively charged (acidic) side chain, which energetically destabilizes the protein structure.

The V411E substitution occurring in *nhr-49(et13)* affects an evolutionarily conserved amino acid. Thus, we studied the biophysical consequences of *et13(V411E)* on all NHR-49 isoforms to assess the structural, and consequently predicted functional, disruption, as measured by energetic analysis. Interestingly, the V411E mutation modifies the structure of the loop between two helices and causes an enthalpy change, which is isoform dependent. Specifically, the mutation is predicted to have neutral to moderately stabilizing effects on isoforms B, D, and E (Fig 3A and 3B; S2 Table). Importantly, regardless of impact on protein structure in the static conformation, these two helices represent helix 10 and helix 12 of the conserved NHR structure, which is a dynamic and flexible molecular switch known to change conformation upon NHR transition from transcriptional repressor to activator [37]. As such, the *et13(V411E)* mutation (as well as the *et8(S432F)* and *et7(P479L)* mutations) is anticipated to perturb NHR-49 dependent activation and/or repression by interfering with NHR-49’s capacity to adopt an activating or repressing conformation and, possibly, interaction with MDT-15 and/or other interaction partners (Fig 3B).

### *nhr-49* gof mutations broadly activate NHR-49 target genes

**Fig 4:**
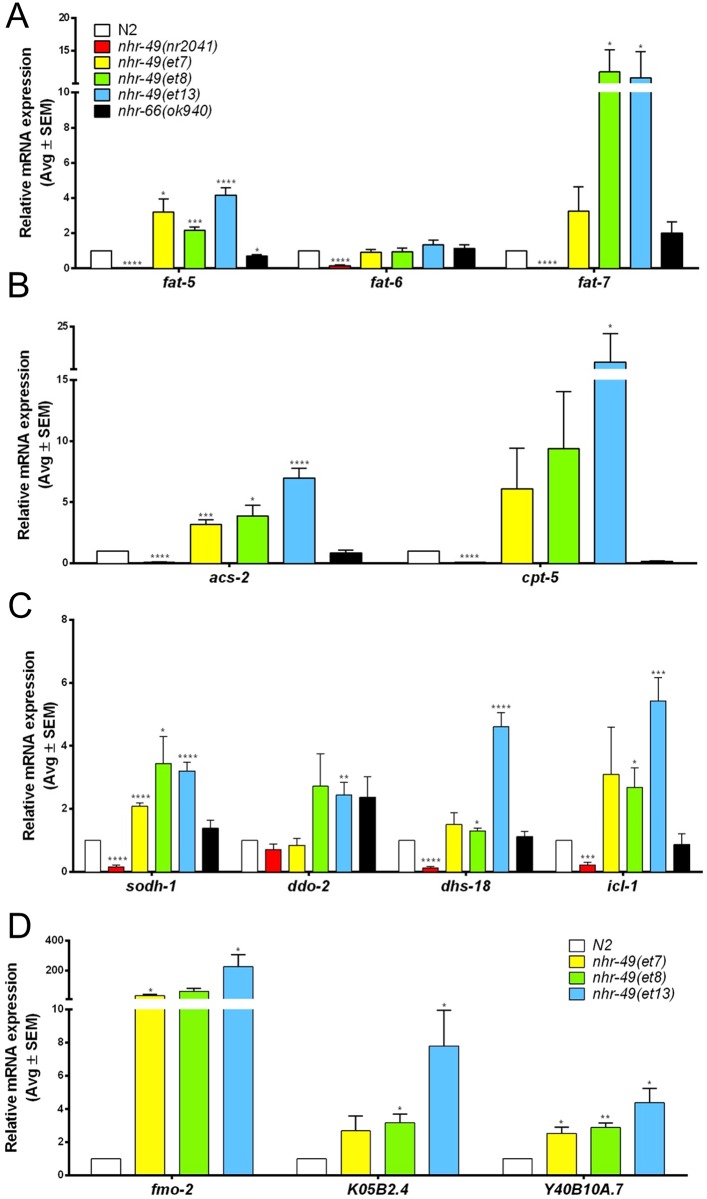
*nhr-49* gof strains broadly affect *nhr-49* dependent activated genes. Bar graphs show average mRNA fold-changes (*vs*. wild-type) in L4 stage wild-type N2 worms, *nhr-49(nr2041)* and *nhr-66(ok940)* null mutants, and *nhr-49(et7)*, *nhr-49(et8)*, and *nhr-49(et13)* gof mutants (n ≥ 3). Gene expression normalized to *act-1*, *tba-1*, and *ubc-2*. Errors bars show SEM. *, p<0.05; **, p<0.01; ***, p<0.001; and ****, p<0.0001 (unpaired Student's *t*-test). (A) Fatty acid desaturase genes. (B) Fatty acid β-oxidation enzymes. (C) Non-lipid metabolism genes. (D) Stress response genes.

Having established that the three missense mutations in the NHR-49-LBD likely affect biophysical properties, we next wished to evaluate their impact on transcription. All three gof alleles originate from the same cold sensitivity suppressor screen and presumably compensate for the *paqr-2* mutation by the same mechanism, i.e. by increasing membrane fluidity through the upregulation of unsaturated fatty acid levels via transcriptional activation of the fatty acid desaturase *fat-7* [25]. However, this model has only been ascertained for *et8(S432F)*, and only in the *paqr-2/adiponectin receptor* mutant background, which itself might cause the deregulation of some lipid metabolism genes [25,38]. To gain better insight into the gene expression changes caused solely by the gof mutations, we outcrossed them to wild-type worms, removing the *paqr-2* mutation. In the resulting gof single mutant strains, we performed real-time quantitative PCR (qPCR) analysis of NHR-49 activated genes. We used *nhr-49(nr2041)* null mutants as a positive control. We also assessed gene expression in *nhr-66(ok940)* null mutants, as NHR-66 is an NHR-49 dimerization partner that appears to exclusively affect NHR-49 repressed genes [13]. As expected, all tested NHR-49 activated genes were downregulated in the *nhr-49(nr2041)* null mutants but largely unaffected in *nhr-66(ok940)* null mutants (Fig 4).

First, we studied the three fatty acid desaturase genes and NHR-49 targets *fat-5*, *-6*, and *-7* [15] (Fig 4A). In line with previously published data [25], *nhr-49(et8)* mutants showed a significant upregulation of *fat-7*, and also induced *fat-5* expression. *nhr-49(et13)* worms showed similar changes, whereas the effects were less pronounced in *nhr-49(et7)* worms (significant only for *fat-5*, but not for *fat-7*). In contrast, we observed no significant upregulation of *fat-6*, in agreement with published findings that *fat-6* dependence on *nhr-49* is much weaker than that of *fat-5* and *-7* [14,15,29]. Collectively, these data are consistent with the model that the *nhr-49* gof alleles increase the levels of unsaturated fatty acids by upregulating fatty acid desaturase genes.

Besides fatty acid desaturases, NHR-49 also affects fatty acid β-oxidation enzymes and non-fatty acid metabolism genes [13–15]. To test whether the effects of *nhr-49* gof alleles were restricted to fatty acid desaturase genes or broadly affect NHR-49 targets, we next assessed the expression of β-oxidation enzyme genes *acs-2* and *cpt-5*, and non-fatty acid metabolism genes *sodh-1*, *ddo-2*, *dhs-18*, and *icl-1* (Fig 4B and 4C). Most genes were induced or trended towards induction in all three gof strains, with *nhr-49(et13)* generally showing the strongest activations and *nhr-49(et7)* displaying the weakest inductions. Lastly, we also found that all three gof strains induced several stress response genes [39,40] (Fig 4D). We conclude that, although identified in a screen for suppressors of cold sensitivity, the *nhr-49* gof mutations do not only affect the fatty acid desaturase genes known to impact membrane fluidity and low temperature adaptation, but also broadly promote the expression of NHR-49 activated genes.

### The *et13(V411E)* substitution causes de-repression of NHR-49 repressed genes

**Fig 5:**
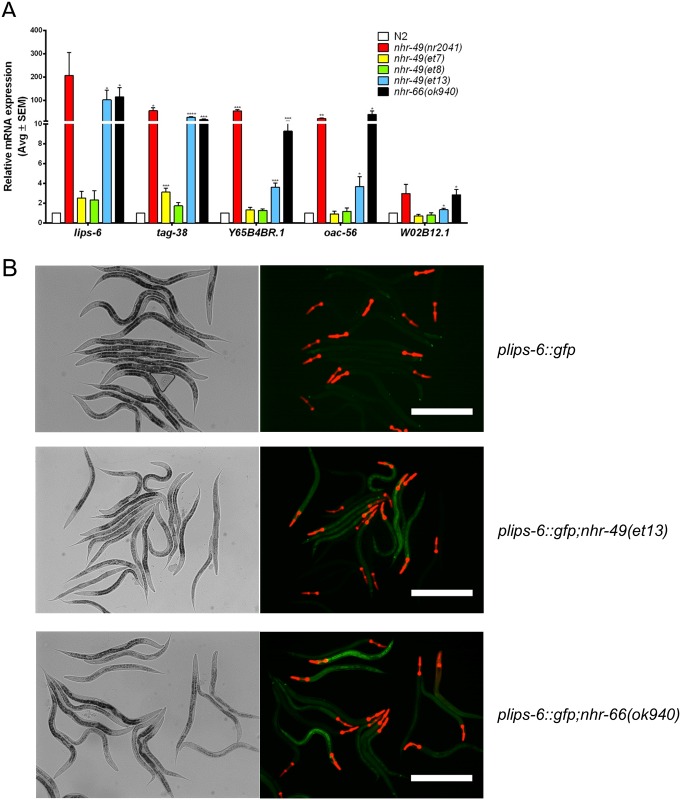
Selective upregulation of NHR-49 repressed genes in *nhr-49(et13)* mutants. (A) Bar graphs show average mRNA fold-changes (*vs*. wild-type) of sphingolipid breakdown and lipid metabolism genes in L4 stage wild-type N2 worms, *nhr-49(nr2041)* and *nhr-66(ok940)* null mutants, and *nhr-49(et7)*, *nhr-49(et8)*, and *nhr-49(et13)* gof mutants (n ≥ 3). Gene expression normalized to *act-1*, *tba-1*, and *ubc-2*. Errors bars show SEM. *, p<0.05; **, p<0.01; ***, p<0.001; and ****, p<0.0001 (unpaired Student's *t*-test). (B) DIC and fluorescence micrographs show *plips-6*::*gfp* worms in wild-type, *nhr-49(et13)*, and *nhr-66(ok940)* worms. Size bar 200 μm.

NHR-49 also represses certain genes [13]. Thus, we studied the expression of NHR-49 repressed genes in the three gof strains, expecting that they might be unaltered (if gof mutations affected solely NHR-49’s activation function) or repressed (if the gof alleles enhanced NHR-49’s repressive function). In line with published data [13], the predicted sphingolipid breakdown gene *tag-38/sphingosine-1-phosphate lyase 1* and the predicted lipid metabolism enzymes *lips-6/lipase*, *oac-56/O-acyltransferase*, Y65B4BR.1/phospholipase B1, and W02B12.1/phospholipase B1 were strongly induced in *nhr-49(nr2041)* and *nhr-66(ok940)* null mutants (Fig 5A). Surprisingly, whereas *et7(P479L)* and *et8(S432F)* only very weakly affected *lips-6* and/or *tag-38* and displayed unaltered expression of the other tested genes, *nhr-49(et13)* mutants displayed a significant upregulation of *lips-6*, *tag-38*, Y65B4BR.1, *oac-56*, and W02B12.1 (Fig 5A). In the case of *lips-6* and *tag-38*, this induction was as strong as that observed in *nhr-49(nr2041)* and *nhr-66(ok940)* null mutants (Fig 5A).

To corroborate the qPCR data, we generated a transgenic *C*. *elegans* strain carrying a transcriptional gfp reporter for *lips-6*. We used the 2kb region upstream of the predicted transcriptional start site that likely reflects the putative *lips-6* promoter (see Materials and Methods). At the L4 stage, expression of this reporter was very faint or completely absent in wild-type worms (Fig 5B). In contrast, although there was some variation of fluorescence signal between individual worms, *plips-6*::*gfp* expression was evident when the transgene was crossed into the *nhr-66(ok940)* null or the *nhr-49(et13)* gof backgrounds (Fig 5B). Notably, we observed expression in the intestine, a tissue known to express *nhr-49* and many metabolic genes [15,18]. Taken together, based on the upregulation of NHR-49 repressed genes including *lips-6* in the *nhr-49(et13)* strain, we conclude that the *et13(V411E)* mutation likely causes a dual loss- and gain-of-function.

### The gof mutations do not alter NHR-49 expression or subcellular localization

**Fig 6:**
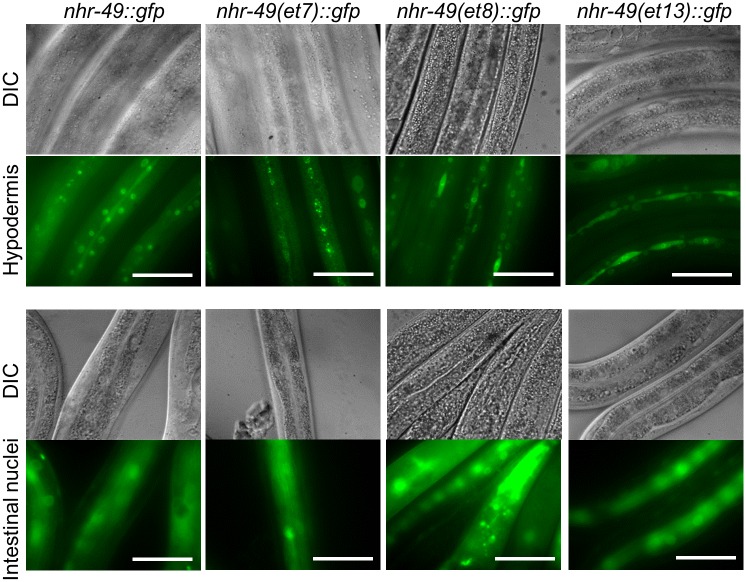
The gof mutations do not affect overall expression and subcellular localization of NHR-49. DIC and fluorescence micrographs show NHR-49::GFP fusion proteins in in hypodermis and seam cells (top panel) and intestine (bottom panel) of the NHR-49::GFP, NHR-49(et7)::GFP, NHR-49(et8)::GFP, and NHR-49(et13)::GFP. Size bar 50 μm.

To determine possible mechanisms leading to increased transcriptional activity, we next tested whether the gof mutations affect NHR-49 protein levels and/or localization. To date, no nuclear localization signal or nuclear export signal has been defined in NHR-49, and computational NLS prediction revealed only a low-confidence NLS in the N-terminus of NHR-49. To test whether the gof mutants affect NHR-49 protein levels or subcellular distribution, we generated transgenic strains expressing each individual gof mutation within a translational NHR-49::GFP fusion protein and compared them to wild-type NHR-49::GFP [18]. We found that NHR-49(et7)::GFP, NHR-49(et8)::GFP, and NHR-49(et13)::GFP showed strong similarity to wild-type NHR-49::GFP: they were expressed at similar overall levels, and in similar patterns in nuclei and cytoplasm of intestinal cells, seam cells, and the hypodermis (Fig 6; S1 Fig). Thus, the gof alleles are unlikely to achieve their effects through altered NHR-49 expression and/or localization.

### Differential effects of the gof mutations on ligand-protein interactions *in silico*

**Fig 7:**
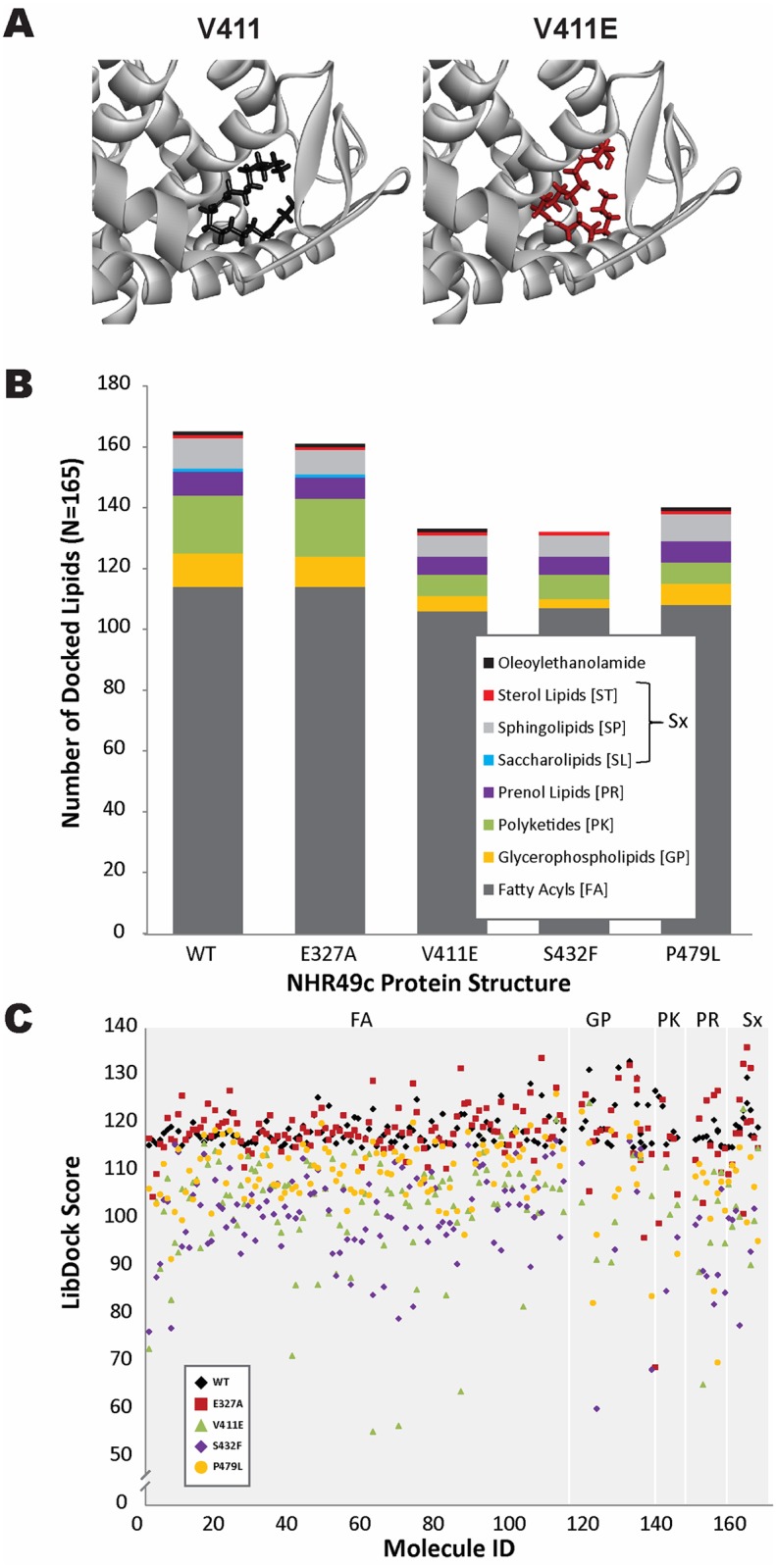
Computational docking of fatty acid ligands to wild-type and mutant NHR-49 LBDs. (A) Molecular docking of oleoylethanolamide in the NHR-49C WT LBD (black) and the V411E gof LBD (red). The orientation of the long chain lipid molecule is influenced by the structural modifications resulting from the missense mutation. (B) Total number of lipid molecules docked for each NHR-49 mutation (LibDock scores >115) by class. The gof mutations are restricted in both the number and nature of lipid ligands in the modified LBDs compared with the WT or the E327A dimerization mutant. (C) Comparison of the LibDock scores quantifying the energetics and interactions of the lipid ligand (a higher score indicates more favorable binding) within the mutant protein structures by class (FA = fatty acyls; GP = glycerophospholipids; PK = polyketides; PR = prenol lipids; Sx = sterol lipids (ST), sphingolipids (SP), saccharolipids (SL)). Note that ligands that cannot dock within mutant LBDs are not represented as no LibDock score is generated.

Besides affecting NHR-49 level or localization, the gof alleles might alter ligand and/or protein binding, as all three gof mutations afflict residues situated in or near NHR-49’s LBD. Notably, the HNF4 residue orthologous to NHR-49 V411, which is mutated in *nhr-49(et13)*, helps coordinate the binding of a fatty acid ligand [34,36]. This suggests that *et13(V411E)* may alter ligand binding in NHR-49. No ligand has yet been identified for NHR-49. Thus, we employed unbiased *in silico* screening of a library comprised of 6,313 lipid molecules (<C19) to assess the functional impact of gof substitutions on ligand binding relative to the wild-type protein. Initial ligand docking experiments in the NHR-49C homology model yielded >450,000 poses for 5,654 lipids docked in the LBD binding site (see Materials and Methods for details). LibDock scores ranged from 29.065 (C_3_H_6_O short chain fatty acyl) to 133.845 (C_17_H_33_O_7_P glycerophosphates). For example, oleoylethanolamide, which binds NHR-80, an NHR-49 dimerization partner also related to HNF4 [19], had a LibDock score of 119.228 (Fig 7A). These data suggest that more complex, longer chain lipid molecules (C14-C21) may be the preferential ligands for NHR-49, with fatty acyls and polyketides having the highest ranked scores. Using the top-ranked lipid ligands (LibDock score >115) from the WT model, a subset of ligands (n = 165) was compared across the four mutant models, E327A, V411E, S432F, and P479L (Fig 7A; see S3 Table for docking scores of the top 165 ligands). The structural modifications resulting from the mutations caused a number of lipid ligands to fail docking, with a maximum of 163 ligands docked for E327A and a minimum of 132 lipids docked for S432F (Fig 7B). Intriguingly, V411E and S432F mutations failed to interact with 26 ligands, 19 of which also failed to dock in the P479L model. Moreover, even with successful docking, the energetics of the lipid:protein interaction, nature and number of bonds formed, and the resulting LibDock score varied widely for a given lipid. Notably, proteins encoding a gof allele generally trended towards reduced ligand binding capacity (Fig 7C). Specific classes of lipids showed substantial differences in their predicted ability to interact with WT or NHR-49(gof) proteins. In particular, saccharolipids completely failed to dock in any of the NHR-49(gof) mutant proteins. Similarly, fewer polyketides and glycerophospholipids were able to dock the LBDs of NHR-49(gof) proteins than that of WT NHR-49. In contrast, the modeled interactions of WT NHR-49 with target ligands were largely maintained in the NHR-49-E327A mutant model (n = 165 *vs*. 163). This demonstrates that loss-of-function mutations in the dimerization region have limited influence on the LBD’s ligand binding capacity, as expected.

### Differential effects of the gof mutations on protein-protein interactions

**Fig 8:**
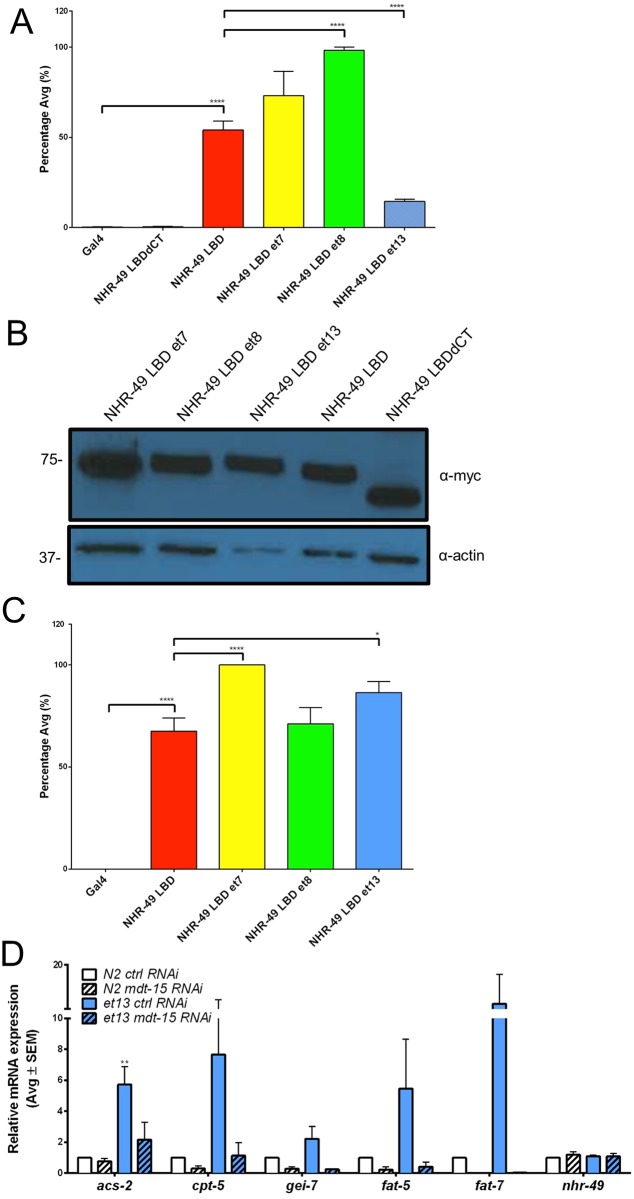
Effects of NHR-49 gof mutations and of C-terminal truncation on physical interaction with MDT-15 and NHR-66. (A-C) Bar graphs indicate relative interaction strength between Gal4DBD-NHR-49 variants and Gal4AD-MDT-15 (A) or Gal4AD-NHR-66 (C). Values indicate average interaction strength in percent, calculated from Miller units (n>4 per plasmid combination); error bars represent SEM. *, p<0.05; ****, p<0.0001 (unpaired Student’s *t*-test). (B) Western blot analysis against the Myc-tag of Y2H bait plasmids demonstrates similar expression levels of WT and gof Gal4-DBD-NHR-49 baits; α-actin served as loading control; full immunoblots are shown in S2 Fig. (D) *nhr-49(et13)* requires *mdt-15* to express *nhr-49* activated genes. Bars represent average mRNA levels of *nhr-49* activated genes in synchronized, L4 stage wild-type N2 and *nhr-49(et13)* gof worms grown on *control* RNAi (empty vector) or on *mdt-15* RNAi (n = 3). RNA levels were normalized to *act-1*, *tba-1*, and *ubc-2*. Errors bars show SEM. ** represents p<0.01 (unpaired *t*-test).

Besides ligand binding, the LBD also serves as a docking site for NHR coregulators and as a surface enabling NHR dimerization [9,10]. Indeed, the NHR-49 LBD is sufficient and necessary for interaction with the coactivator MDT-15, for homodimerization, and for heterodimerization with other NHRs, including NHR-66 [13,29,40]. We hypothesized that the gof mutations might potentially increase interaction with the coactivator MDT-15 and, for *et13(V411E)*, possibly decrease binding to the repressive dimerization partner NHR-66. To test these hypotheses we used the yeast-two-hybrid (Y2H) system. To study the effects of the gof point mutations, we introduced each mutation individually by site-directed mutagenesis into the NHR-49-LBD bait, and assessed the effect on MDT-15 and NHR-66 prey binding. As expected [29], the wild-type NHR-49-LBD bait (aa 130–501) strongly bound the MDT-15 prey (Fig 8A; note that NHR-49-LBD baits bearing gof substitutions were expressed at levels similar to wild-type NHR-49-LBD, Fig 8B). Compared to this interaction, the *et8(S432F)* substitution caused a significant, but modest increase in MDT-15 binding, whereas the *et13(V411E)* substitution unexpectedly reduced MDT-15 binding (Fig 8A). Additionally, the *et7(P479L)* and *et13(V411E)* substitutions weakly but significantly increased NHR-66 binding (Fig 8C). Thus, *et8(S432F)* may cause gof by increasing NHR-49 binding to MDT-15, whereas *et13(V411E)* substitution apparently achieves gof and lof effects despite decreased MDT-15 binding and increased NHR-66 binding.

As *et13(V411E)* causes severely reduced MDT-15 binding, we hypothesized that it might have lost *mdt-15* dependence, with the gof effect arising from neofunctionalization, i.e. the acquisition of a molecular function not present in wild-type NHR-49. To test this hypothesis, we performed *mdt-15* depletion by RNAi interference in wild-type N2 and *nhr-49(et13)* gof worms. The expression of NHR-49 target genes activated in *nhr-49(et13)* remained dependent on *mdt-15* (Fig 8D). We conclude that although MDT-15:NHR-49 binding is reduced by the *et13(V411E)* allele, MDT-15 remains a critical coactivator in the *nhr-49(et13)* strain.

*et7(P479L)* resides in the C-terminal extension that is only conserved in NHR-49 orthologs from *Caenorhabditis* species, and is adjacent to a predicted 9aaTAD motif (Fig 1). We speculated that the C-terminal extension of NHR-49’s LBD might be required for MDT-15 binding. Indeed, C-terminal truncation of the LBD (NHR-49dCT, aa 130–434 instead of aa 130–501) completely abrogated MDT-15 binding (Fig 8C), whereas NHR-49-LBD bait expression was not substantially affected (Fig 8B). Thus, the C-terminal extension harboring *et7(P479L)* is required for MDT-15 binding, at least in the Y2H system.

### *nhr-49* gof mutations differentially affect life span

**Fig 9:**
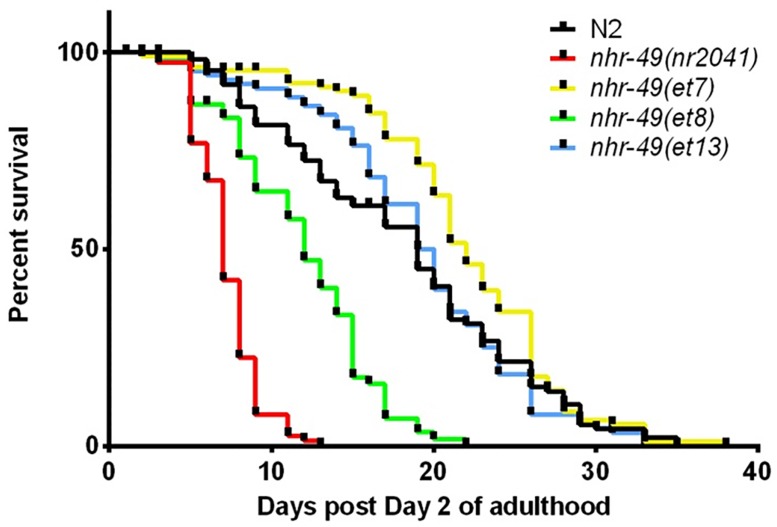
*nhr-49* gof alleles differentially affect worm life span. Population survival curves of wild-type N2 worms and *nhr-49(et7)*, *nhr-49(et8)*, and *nhr-49(et13)* gof mutants. One of three to four individual experiments with similar outcomes is shown; see S4 Table and S3 Fig for details on replicates and statistical analysis. All lifespan experiments were performed at 20°C.

In the *paqr-2* mutant background, strains carrying *nhr-49* gof mutations display increased viability and brood size at 16°C as well as amelioration of a morphological phenotype [25]. We wondered whether the gof mutations would affect any other phenotype linked to *nhr-49*. Specifically, because *nhr-49* null mutation or depletion results in a shortened lifespan [15,18,29], we tested whether strains carrying *nhr-49* gof mutations display altered population survival, expecting an increase in average lifespan. Interestingly, we found that the three strains displayed distinct phenotypes: *nhr-49(et7)* was long-lived, *nhr-49(et8)* was short-lived, and *nhr-49(et13)* displayed a wild-type life span (Fig 9). Thus, the three *nhr-49* gof mutations evoke distinct, non-equivalent effects on animal life span.

## Discussion

*C*. *elegans* NHR-49 is a sequence ortholog of HNF4 and an important regulator of lipid metabolism and life span [13–15,18,19,23,40]. Svensk *et al*. previously reported three *nhr-49* gof mutations that suppress the cold sensitivity and a morphological phenotype of the *paqr-2/adiponectin receptor* mutant [25]. The fact that three distinct *nhr-49* mutations were isolated provided an opportunity to learn about how individual residues determine NHR-49’s transcriptional activity. Using molecular and computational methods, we found that all three gof alleles likely achieve suppression of cold sensitivity through the same mechanism, namely induction of fatty acids desaturation. In contrast, the three alleles are non-equivalent in regards to gene expression and phenotypes not related to this condition. Most notably, *nhr-49(et13)* is qualitatively different from *nhr-49(et7)* and *nhr-49(et8)*. Mechanistically, the protein interaction and subcellular localization studies did not identify clear molecular mechanisms of action for the three mutations. However, our computational modeling suggests that the gof missense mutations may alter ligand-binding abilities compared to WT, providing a possible mechanism of action.

In the present study we set out to better characterize three *nhr-49* gof mutations. We especially attempted to determine whether the residues they alter represent putative specificity determinants, i.e. whether they lie within surfaces or functional regions that allow NHR-49 to specifically regulate certain downstream processes (e.g. fatty acid desaturation *vs*. fatty acid β-oxidation). As expected due to the origin of these mutations from a suppressor screen for cold-sensitivity [25], we observed induction of *fat-5* and/or *fat-7* by all three gof alleles. Other NHR-49 activated genes were not as consistently activated, with e.g. *et7(P479L)* failing to induce *ddo-2* and *dhs-18*, and *et13(V411E)* consistently causing stronger activation than the other two alleles. Collectively, these data suggest that the three *nhr-49* gof alleles differ at the very least in how strongly they induce NHR-49 activated genes. Additionally, we observed a striking difference in *nhr-49*-dependent repression, which was largely unaltered by *et7(P479L)* and *et8(S432F)* but at least partially abrogated by *et13(V411E)*. Although identified as a gof allele, *nhr-49(et13)* thus also displays some loss-of-function characteristics, rendering this allele qualitatively distinct from the other two. From these studies we conclude that V411 is essential for both NHR-49 driven activation and repression whereas P479 and S432F represent residues that are specific to gene activation. Unbiased expression profiling by mRNA sequencing will substantiate this notion and should also reveal whether any of the gof mutations affect the expression of genes not regulated by wild-type NHR-49.

In line with the quantitative and qualitative differences in gene expression, we found that the three mutant worms strains were non-equivalent with regards to a phenotype investigated, namely life span. Specifically, *nhr-49(et7)* worms are long-lived, *nhr-49(et8)* worms are short-lived, and *nhr-49(et13)* mutants display a normal life span. The differential effects of the gof mutations on a phenotype are distinct from the effects observed previously: Svensk *et al*. showed that, in the *paqr-2* mutant background, all three *nhr-49* gof alleles are similarly capable of ameliorating the cold sensitivity and withered tail phenotype [25]. At this time we do not understand what underlies the diversity of longevity phenotypes caused by the three alleles. Perhaps, the comparably mild gene activation in *nhr-49(et7)* mutants resembles that seen in wild-type worms overexpression NHR-49, a genetic change sufficient to induce a long life span [18]. In contrast, the more substantive and/or qualitatively different changes caused by *et8(S432F)* and *et13(V411E)* might counteract any pro-longevity changes in gene expression, e.g. that of increased fatty acid desaturase expression [13,41,42], to the extent that the ultimate effect is neutral (for *et13(V411E)*) or even negative (for *et8(S432F)*).

We attempted to delineate how, molecularly, the three substitutions affect NHR-49 activity. All three affected residues are situated within or near NHR-49’s LBD, a domain critical for transcriptional regulation Thus, we hypothesized that interactions with molecular partners such as the coactivator MDT-15 [29] or the dimerization partner NHR-66, involved in NHR-49 dependent repression [13], might be altered. Surprisingly, NHR-66 interaction was only modestly affected and MDT-15, although bound less strongly by NHR-49(et13), remained critical for gene activation in worms expressing this *nhr-49* mutant protein. We also failed to detect major changes in NHR-49 protein expression and localization, as determined by translational reporter analysis. It is possible that putative effects of the substitutions on the protein are masked in these experiments relying on extrachromosomal arrays, which result in NHR-49 overexpression; thus, endogenous NHR-49, which is not studied by this approach, may be expressed and/or localized differently in the gof strains. Nevertheless, based on our studies we consider it likely that the three gof alleles appear to increase NHR-49 activity through mechanisms other than changing protein abundance, nuclear accumulation, or interaction with MDT-15. Future experiments such as Y2H screens using point mutant NHR-49-LBDs as bait may help identify mechanisms underlying the increased transcriptional activity.

The LBD is also the binding site for ligand. Because no ligand is yet known for NHR-49, we attempted to delineate the potential influence of the gof missense mutations by *in silico* ligand docking. To our knowledge this is the first attempt of computational modeling and ligand docking of a *C*. *elegans* HNF4α-like NHR, and this methodology strongly suggests that NHR-49 adopts a structure resembling HNF4α, as expected based on the sequence homology [9]. This approach did not identify any particular class of molecules that could explain the allele-specific phenotypes or the *et13(V411E)*-specific gene repression defects. However, our modeling suggested that the proteins produced by the gof alleles lack the ability to bind saccharolipids, polyketides, and glycerophospholipids. Either of these changes could promote gene activation by NHR-49 missense mutants; in particular, polyketides are of interest as they bind and/or regulate NHRs such as human PPARs and PXRs [43–45]. Thus, it is possible that reduced binding of such molecules causes the increased gene activation observed in strains bearing *nhr-49* gof mutations. Moreover, it is possible that such molecules might represent ligands for other, NHR-49-related NHRs of *C*. *elegans*.

The most striking observation we made was the surprising finding that NHR-49 repressed genes were unexpectedly upregulated in the presumed gof mutant *nhr-49(et13)*. Our structural homology modeling predicts that the V411E mutation substantially alters the biophysical properties of this residue. Notably, V411 is part of helix 12, and thus constitutes an important part of the repressor-to-activator switch in the classical NHR LBD. The mixed gene expression phenotypes of *nhr-49(et13)*, with both strong activation and de-repression, suggest that V411 is primarily required for repression. In contrast, activation can still be successfully achieved in this mutant. It is possible that this is due to multi-surface contact with coactivators such as MDT-15 through the C-terminal extension including the 9aaTAD motif near P479. No corepressor has yet been identified for NHR-49, and only two corepressor have been described for *C*. *elegans* NHRs to date, DIN-1 and GEI-8 [46,47], none of which appear to influence *nhr-49* repressed genes (KL and ST, unpublished). The transcriptional *lips-6* reporter described in this study should be a useful tool to identify candidate NHR-49 (and NHR-66) corepressors in the future using forward or reverse genetic screens.

In sum, our analysis shows that, although they were identified in the same screen and similarly affected the phenotype used as a readout in said screen, the three *nhr-49* gof mutants display qualitative and quantitative differences in gene expression and phenotypes, likely by influencing distinct molecular properties of NHR-49.

## Materials and Methods

### Sequence alignments and motif prediction

Predictions of nematode NHR-49 orthologs were from WormBase WS248 and/or a BLASTP search with NHR-49 isoform C. Full-length sequences for these NHR-49 orthologs were downloaded from UniProt (http://www.uniprot.org/). Sequence alignments were performed using ClustalW (http://www.genome.jp/tools/clustalw/). 9aaTAD motifs were detected using the online prediction tool (http://www.med.muni.cz/9aaTAD/index.php) [30,31]. We selected the “most stringent pattern” option to identify 9aaTAD motifs in NHR-49 isoform C. NLS prediction was performed with cNLS Mapper (http://nls-mapper.iab.keio.ac.jp/cgi-bin/NLS_Mapper_form.cgi) [48].

### *In silico* structural modeling and docking of NHR-49

All *in silico* analysis was performed using Accelrys DiscoveryStudio v4.1 (Biovia). The protein sequences for the five NHR-49 isoforms were imported from NCBI (https://www.ncbi.nlm.nih.gov/protein) and sequence aligned. Using the built in BLAST search algorithm (http://blast.ncbi.nlm.nih.gov/Blast.cgi), three high confidence crystal structures for HNF4 from RCSB (http://www.rcsb.org/) were identified (PDBs 4IQR; 1LV2; 1M7W) and the individual isoform amino acid sequences were threaded onto the HNF4 sequence using a Blosum62 scoring matrix. For PPARα and PPARγ, we identified one high confidence crystal structure each (PDB 2REW and PDB 3E00, respectively) that were used. The MODELLER algorithm was used to generate homology models (>10,000 permutations) using the Discrete Optimized Protein Energy (DOPE) method to refine loops. Isoform models were then refined, subjected to energy minimization (CHARMm), and side-chain rotamers were verified. *In silico* mutagenesis was performed and Gibb’s free energy (kcal/mol) was calculated for individual mutant models. The lipid ligand structures were obtained from http://www.lipidmaps.org/data/structure/ using < = C18 as the ontology search and downloaded as.sdf files and prepared for docking at physiological pH. The ligand-binding site was defined for each prepared homology model using coordinates from the HNF4 crystal structure template (PDB 4IQR). High throughput screening of the wild-type and mutant protein models was performed using LibDock [49,50]. LibDock is a rigid docking algorithm in which the protein template is held in constant conformation while the molecular ligand is allowed limited flexibility to generate novel conformations based on atomic, polar, and apolar interactions within the protein cleft. After docking, a final optimization step identifies steric clashes followed by an arbitrarily scored ranking of every potential conformation (aka poses) within the protein target using pairwise atomic clustering and comparative scoring. This involves bond interaction analysis, which prioritizes and refines hydrogen bonding. Similar to a piecewise linear potential the docking score is generated by summation of potential energetics of interacting atoms in the protein-ligand complex relative to bond distance. The atoms are divided into four atom types–apolar, acceptor, donor, and donor/acceptor–and the score between interacting atoms is scored using either a hydrogen binding potential or a steric potential. More favorable bond interactions and bond distances result in a higher LibDock score. For reference, linoleic acid, considered an endogenous ligand for HNF4α [51], generated a LibDock score of 113.42 when docked onto the HNF4α structure. The subset of NHR-49 ligands analyzed in Fig 7B and S3 Table (with LibDock scores >115) is thus restricted to high-scoring molecules that may represent biologically relevant NHR-49 ligands.

### *C*. *elegans* growth and strains

*C*. *elegans* strains were cultured at 20°C, as described [52]. We used nematode growth medium (NGM)-lite (0.2% NaCl, 0.4% tryptone, 0.3% KH_2_PO_4_, 0.05% K_2_HPO_4_) agar plates seeded with *Escherichia coli* strain OP50 except for feeding RNAi, which was performed using HT115 bacteria. Feeding RNAi was performed as described [40]. The empty vector (negative control) and the *mdt-15* RNAi clone (plate 74, well C09) are from the Ahringer library [53] and were sequenced prior to use.

To generate synchronized worm populations, embryos were isolated by sodium hypochlorite treatment and allowed to hatched overnight on unseeded NGM-lite plates; arrested, synchronized L1 larvae were then grown to the desired stage, as indicated, and growth times were adapted to ensure developmental synchronicity of slow-growing mutants.

The strains wild-type Bristol N2, STE68 *nhr-49(nr2014) I*, and STE69 *nhr-66(ok940) IV* have been described [13,15,52]. We backcrossed QC120 *nhr-49(et7) I; paqr-2(tm3410) III*, QC121 *nhr-49(et8) I; paqr-2(tm3410) III*, and QC126 *nhr-49(et13) I; paqr-2(tm3410) III* (25) to wild-type worms to remove the *paqr-2(tm3410)* mutation, generating strains STE108 *nhr-49(et7) I*, STE109 *nhr-49(et8) I*, STE110 *nhr-49(et13) I*.

To generate transgenic reporter strains STE114 *pnhr-49*::*nhr-49(et7)*::*gfp*, STE115 *pnhr-49*::*nhr-49(et8)*::*gfp*, STE116 *pnhr-49*::*nhr-49(et13)*::*gfp*, and STE111 *plips-6*::*gfp*, 100 ng/μl of each plasmid and 100 ng/μl of co-injection marker *pmyo-2*::*mCherry* and empty vector were microinjected in the gonad. We have not tested whether NHR-49::GFP fusion proteins bearing these gof point mutations are functional but this is likely the case as they are single nucleotide and single amino acid substitutions in the context of the *pnhr-49*::*nhr-49*::*gfp* plasmid, which rescues the short lifespan or *nhr-49* null mutants when expressed as a transgene [18]. The *plips-6*::*gfp* transgene was crossed into *nhr-66(ok940)* and *nhr-49(et13)* mutants, generating STE112 *plips-6*::*gfp;nhr-66(ok940)* and STE113 *plips-6*::*gfp;nhr-49(et13)*.

### Plasmids

We used the Q5 Site-Directed Mutagenesis Kit (New England Biolabs E0554S) to introduce gof mutations into the *pnhr-49*::*nhr-49*::*gfp* plasmid [18]. To generate plasmids that contain *nhr-49(et7)*, *nhr-49(et8)*, and *nhr-49(et13)* mutations, we used primers SP2879/80 (cttgcagctctattggcaattc/agttgctgagagcattcc), SP2881/82 (gctccggtctttttacagcaac/ cgtcgaatgatcattgatgtc), and SP2883/84 (tagcttcaggaggattctctg/tcatttatgtacggattcaaac). Plasmids were sequenced following mutagenesis to ensure presence of mutations and absence of off-target mutations.

We generated the *plips-6*::*gfp* plasmid by amplifying the predicted 2 kb *lips-6* promoter region using primers SP3243/44 (forward primer with PstI restriction enzyme site: atgCTGCAGaaaatacggtatgaattttcatagaac, reverse primer with XmaI restriction enzyme site: atgCCCGGGttttgtgttggtttagaacctgaaat) with iProof^™^ High-Fidelity PCR (Bio-Rad). The PCR product was then cloned using the Zero Blunt TOPO PCR cloning kit (Invitrogen #45–0245), sequenced, and subcloned into pPD95.77 using standard procedures with PstI (New England Biolabs) and XmaI (New England Biolabs) restriction enzymes.

### RNA isolation and quantitative real-time PCR

Total RNA was extracted from developmentally synchronized mid-L4 stage worms as assessed by vulval morphology. RNA isolation and qPCR were performed as described [40]. We used *t*-tests (two-tailed, unequal variance) to calculate statistical significance of gene expression changes between different strains (Gaussian distribution). qPCR primers were designed with Primer3web (bioinfo.ut.ee/primer3/) and tested on serial cDNA dilutions to ensure PCR efficiency. Primer sequences are listed in S5 Table.

### Yeast-two-hybrid assays and immunoblots

Yeast-two-hybrid assays to study protein-protein interactions and immunoblots to ascertain protein expression were performed as described [29,40].

### DIC and fluorescence microscopy

For microscopy, worms were transferred onto 2% (w/v) agarose pads, which contained NaN_3_. We captured images on a CoolSnap HQ camera (Photometrics) attached to a Zeiss Axioplan 2 compound microscope, and used MetaMorph Imaging Software with Autoquant 3D digital deconvolution for image acquisition.

### Lifespan assays

Lifespan assays were conducted as described [29,40]. Day 2 of adulthood was used as t = 0 for lifespan analysis. GraphPad Prism 6 software was used for statistical analysis. P values were calculated using the Log-rank (Mantel-Cox) method. Statistics for all lifespan assays are listed in S4 Table.

## Supporting Information

S1 Fig Replicate micrographs of worms expressing wild-type and mutant NHR-49::GFP translational fusion proteins. DIC and fluorescence micrographs show worms expressing wild-type or gof mutant NHR-49::GFP fusion proteins, as indicated. Size bar 50 μm.
(TIF)

S2 Fig Complete immunoblots showing Y2H bait protein expression. Top: α-Myc immunoblot for Y2H bait fusion proteins; bottom: α-actin immunoblot as loading control; “Ctrl”indicates negative control (untransformed yeast); MDT-15ΔCT, MDT-15NT, and MDT-15KIX indicates three plasmids used in experiments not relevant to Fig 8.
(TIF)

S3 Fig Replicate life span experiments. Population survival curves of wild-type N2 worms and *nhr-49(et7)*, *nhr-49(et8)*, and *nhr-49(et13)* gof mutants. See S4 Table for details on all replicates and statistical analysis. All lifespan experiments were performed at 20°C.
(TIF)

S1 Table Identity and similarity of *C*. *elegans* NHR-49C, human HNF4α, human HNF4γ, and human PPARα protein sequences. The Table lists the percent identity and similarity in the indicated protein comparisons, as determined by CULSTALW alignments. FL = full length; DBD = DNA binding domain; LBD = ligand binding domain.
(DOCX)

S2 Table Energetic impact of mutations on the structural stability of NHR-49 isoforms. (DOCX)

S3 Table Docking scores and identities of top 165 ligands docked for NHR-49 WT and gof variants. (XLSX)

S4 Table Population survival data of mutant strains. (DOCX)

S5 Table Sequences of primers used in this study. List of primers used for qPCR and genotyping. All sequences are displayed in 5’ to 3’ orientation.
(DOCX)
