# Supplementary figures and images for: Gain-of-Function Alleles in Caenorhabditis elegans Nuclear Hormone Receptor nhr-49 Are Functionally Distinct
Source: PLoS One. 2016 Sep 12;11(9):e0162708. doi: 10.1371/journal.pone.0162708 (PMC5019492; doi:10.1371/journal.pone.0162708)

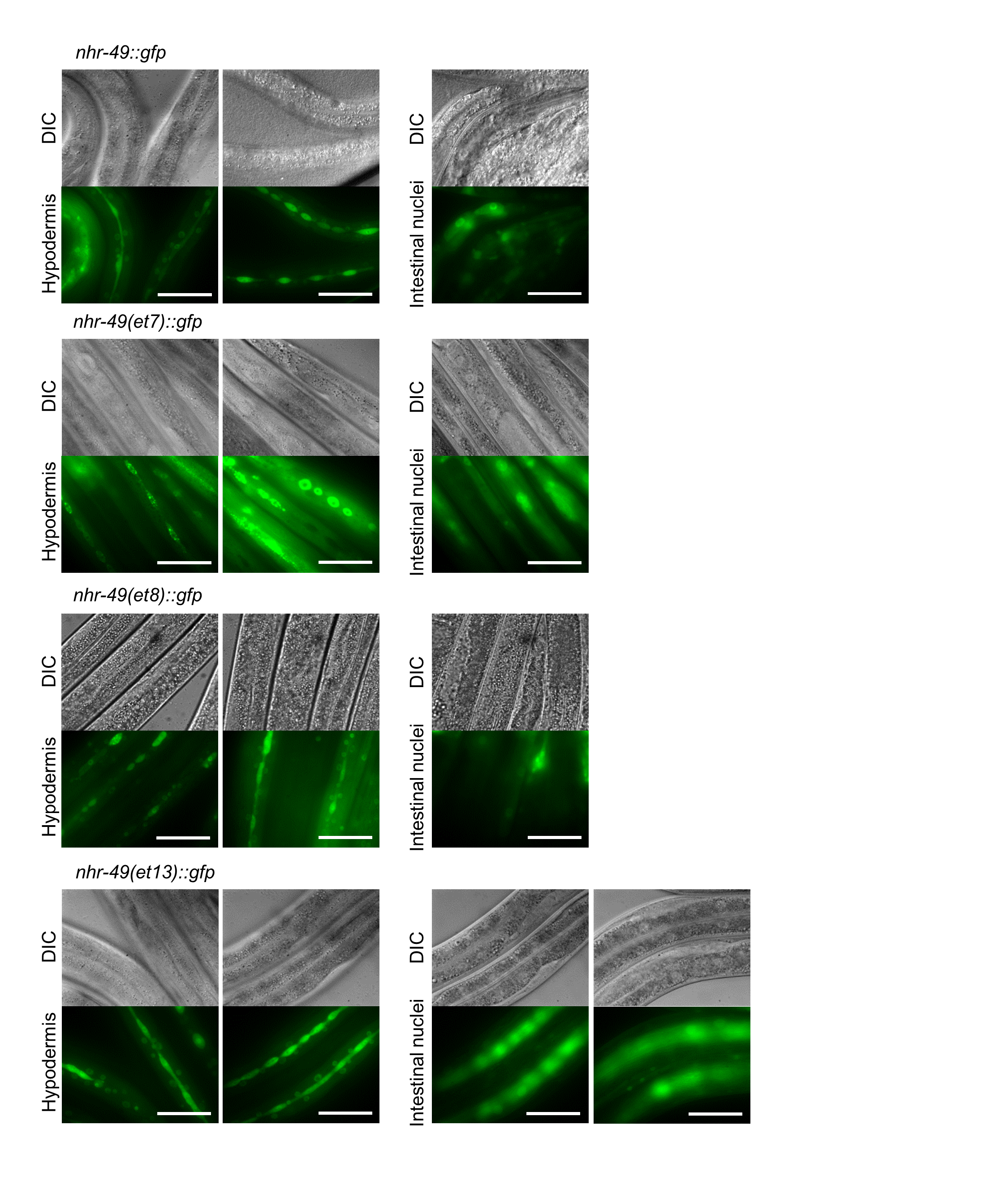

Supplement: S1 Fig — DIC and fluorescence micrographs show worms expressing wild-type or gof mutant NHR-49::GFP fusion proteins, as indicated. Size bar 50 μm. (TIF) [file pone.0162708.s001.tif]

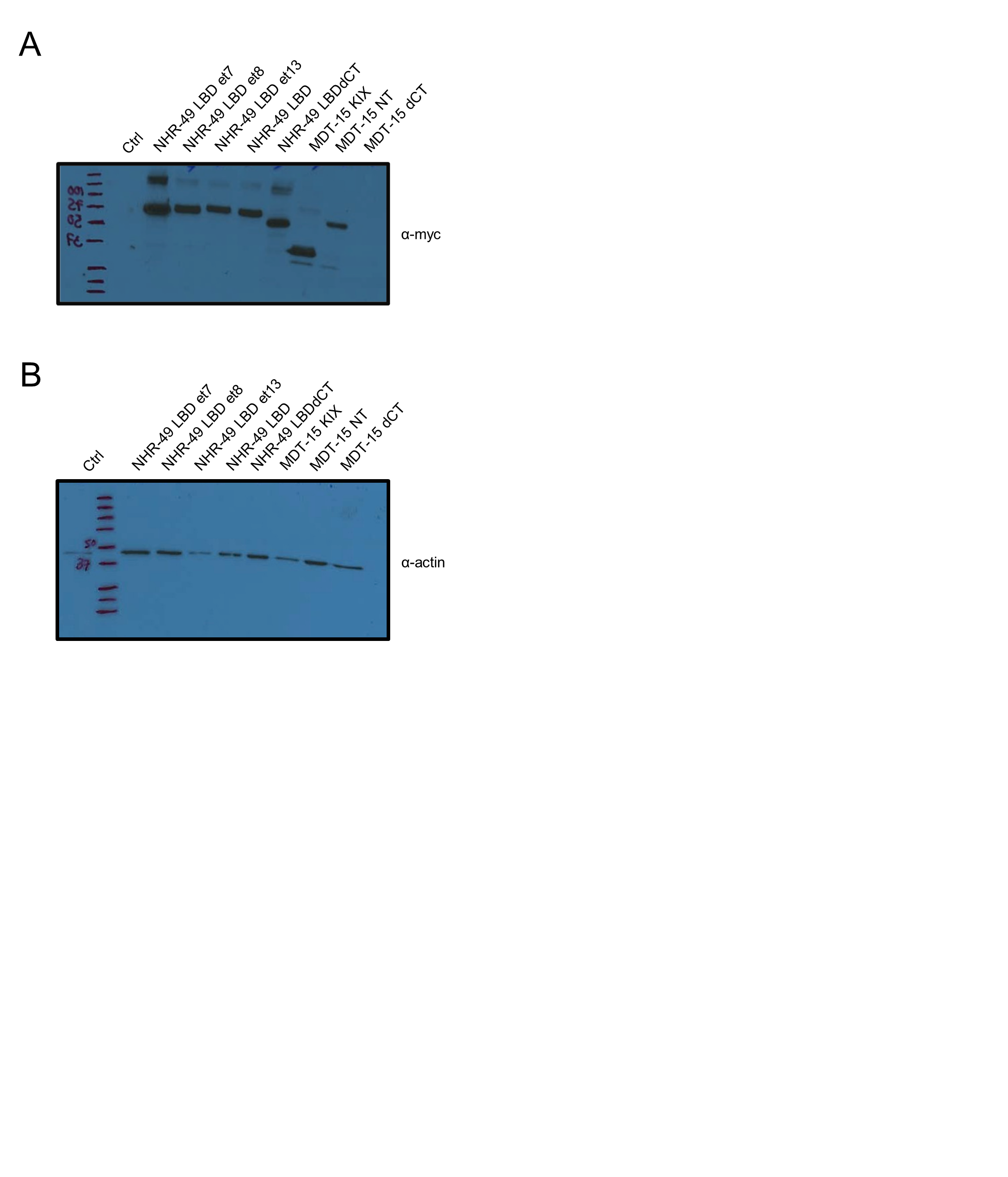

Supplement: S2 Fig — Top: α-Myc immunoblot for Y2H bait fusion proteins; bottom: α-actin immunoblot as loading control; “Ctrl”indicates negative control (untransformed yeast); MDT-15ΔCT, MDT-15NT, and MDT-15KIX indicates three plasmids used in experiments not relevant to Fig 8. (TIF) [file pone.0162708.s002.tif]

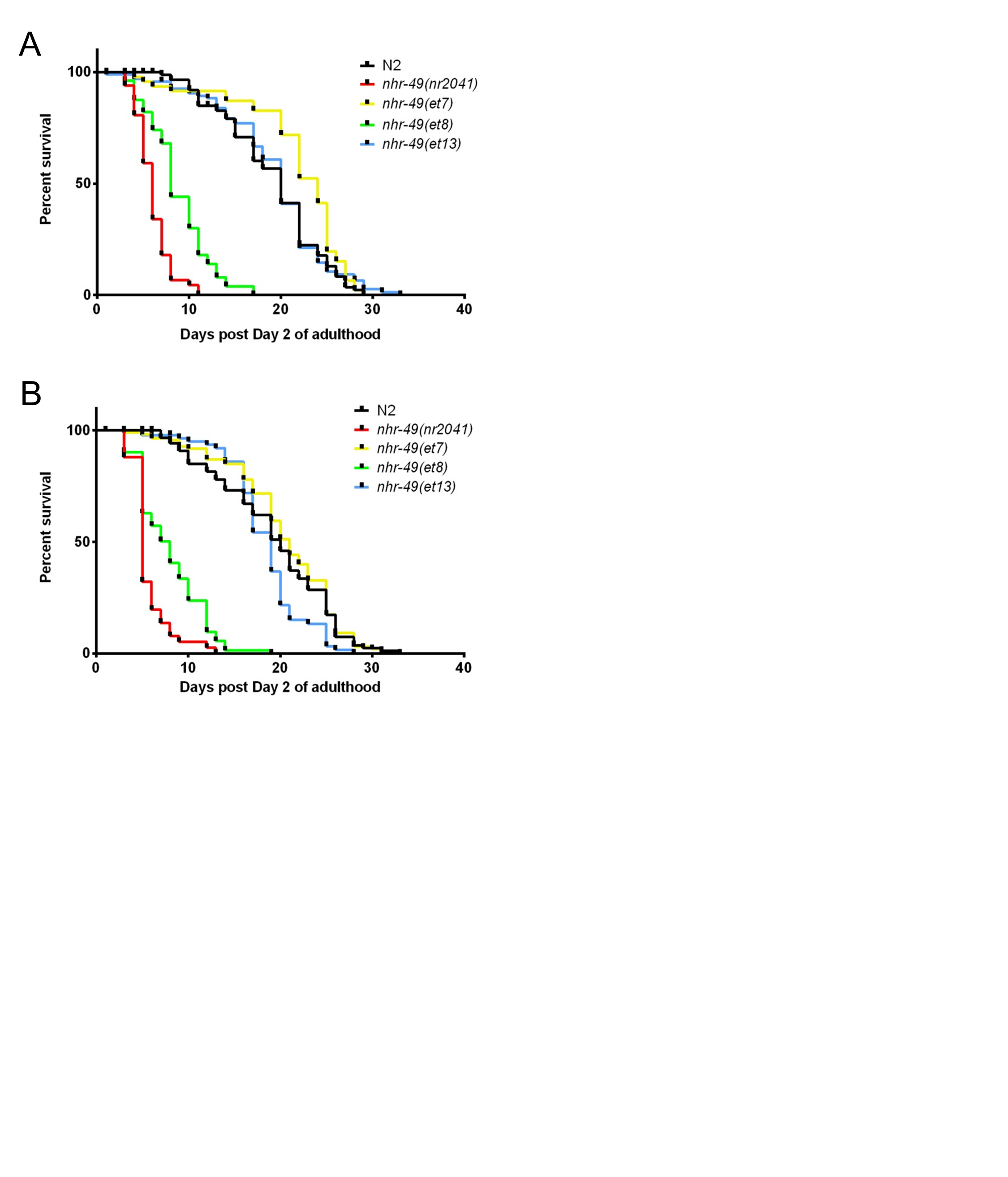

Supplement: S3 Fig — Population survival curves of wild-type N2 worms and nhr-49(et7), nhr-49(et8), and nhr-49(et13) gof mutants. See S4 Table for details on all replicates and statistical analysis. All lifespan experiments were performed at 20°C. (TIF) [file pone.0162708.s003.tif]
