# Supplementary material for: Gain-of-Function Alleles in Caenorhabditis elegans Nuclear Hormone Receptor nhr-49 Are Functionally Distinct
Source: PLoS One. 2016 Sep 12;11(9):e0162708. doi: 10.1371/journal.pone.0162708 (PMC5019492; doi:10.1371/journal.pone.0162708)
Supplement: S1 Table — The Table lists the percent identity and similarity in the indicated protein comparisons, as determined by CULSTALW alignments. FL = full length; DBD = DNA binding domain; LBD = ligand binding domain. (DOCX) [file pone.0162708.s004.docx]

**S1 Table. Identity and similarity of *C. elegans* NHR-49C, human HNF4α, human HNF4γ, and human PPARα protein sequences.** The Table lists the percent identity and similarity in the indicated protein comparisons, as determined by BLASTP alignments. FL= full length; LBD = ligand binding domain; DBD = DNA binding domain.

|  | HNF4α  Identity / similarity | HNF4γ  Identity / similarity | PPARα  Identity / similarity |
| --- | --- | --- | --- |
| NHR-49C FL | 29% / 47% | 29% / 47% | 20% / 31% |
| NHR-49C DBD  (aa 35-110) | 59% / 82%  (aa 60-135) | 58% / 81%  (aa 12-87) | 52% / 68%  (aa 101-184) |
| NHR-49C LBD  (aa 223-417) | 36% / 61%  (aa 151-373) | 36% / 60%  (aa 103-324) | 25% / 49%  (aa 201-467) |
