# Supplementary material for: Gain-of-Function Alleles in Caenorhabditis elegans Nuclear Hormone Receptor nhr-49 Are Functionally Distinct
Source: PLoS One. 2016 Sep 12;11(9):e0162708. doi: 10.1371/journal.pone.0162708 (PMC5019492; doi:10.1371/journal.pone.0162708)
Supplement: S2 Table — (DOCX) [file pone.0162708.s005.docx]

| S1 Table. Energetic impact of mutations on the structural stability of NHR-49 isoforms. | | | | | | | | | |  |
| --- | --- | --- | --- | --- | --- | --- | --- | --- | --- | --- |
| Isoform | NHR-49A |  | NHR-49B |  | NHR-49C |  | NHR-49D |  | NHR-49E |  |
| Mutation | **Mutation Energy (kcal/mol)** | **Effect** | **Mutation Energy (kcal/mol)** | **Effect** | **Mutation Energy (kcal/mol)** | **Effect** | **Mutation Energy (kcal/mol)** | **Effect** | **Mutation Energy (kcal/mol)** | **Effect** |
| E327A | 2.19 | DESTABILIZING | 2.77 | DESTABILIZING | 2.28 | DESTABILIZING | 0.33 | NEUTRAL | 0.39 | NEUTRAL |
| V411E | 1.3 | DESTABILIZING | 0.44 | NEUTRAL | 0.52 | DESTABILIZING | -0.67 | STABILIZING | -0.31 | NEUTRAL |
| S432F | -0.03 | NEUTRAL | -0.32 | NEUTRAL | -0.21 | NEUTRAL | -1.39 | STABILIZING | -0.67 | STABILIZING |
| P479L | -0.71 | STABILIZING | -1.06 | STABILIZING | -2.45 | STABILIZING | -2.96 | STABILIZING | -1.04 | STABILIZING |
