# Supplementary material for: Gain-of-Function Alleles in Caenorhabditis elegans Nuclear Hormone Receptor nhr-49 Are Functionally Distinct
Source: PLoS One. 2016 Sep 12;11(9):e0162708. doi: 10.1371/journal.pone.0162708 (PMC5019492; doi:10.1371/journal.pone.0162708)
Supplement: S4 Table — (DOCX) [file pone.0162708.s007.docx]

**S2 Table. Population survival data of mutant strains.**

Number of subjects is denoted as follows: Nx=total number of animals used in assay; Cx=number of censored events (*i.e.* worms that ruptured at the vulva, underwent internal hatching of the progeny, or crawled off the plate). All *p*-values are derived using the log-rank (Mantel-Cox) test. **p*<0.05, ***p*<0.01, ****p*<0.001, *****p*<0.0001.

| Strain | Experiment no. | Median lifespan (days post day 2 of adulthood) | Number of subjects (Nx (Cx)) | *p*-value *vs.* N2 |
| --- | --- | --- | --- | --- |
| N2 | 1 | 19 | 120(23) | NA |
|  | 2 | 20 | 102(17) | NA |
|  | 3 | 20 | 101(19) | NA |
|  | 4 | 19 | 122(12) | NA |
| *nhr-49 (nr2041)* | 1 | 7 | 124(36) | <0.0001**** |
|  | 2 | 6 | 102(39) | <0.0001**** |
|  | 3 | 5 | 102(34) | <0.0001**** |
| *nhr-49(et7)* | 1 | 22 | 114(22) | 0.0209* |
|  | 2 | 24 | 53(7) | 0.0088** |
|  | 3 | 21 | 122(23) | 0.4659 |
|  | 4 | 21 | 119(19) | 0.0020** |
| *nhr-49(et8)* | 1 | 12 | 118(59) | <0.0001**** |
|  | 2 | 8 | 82(30) | <0.0001**** |
|  | 3 | 8 | 117(40) | <0.0001**** |
| *nhr-49(et13)* | 1 | 20 | 117(28) | 0.9652 |
|  | 2 | 20 | 101(18) | 0.5646 |
|  | 3 | 19 | 104(41) | 0.0298* |
|  | 4 | 19 | 121(25) | 0.8384 |
