# Supplementary material for: Gain-of-Function Alleles in Caenorhabditis elegans Nuclear Hormone Receptor nhr-49 Are Functionally Distinct
Source: PLoS One. 2016 Sep 12;11(9):e0162708. doi: 10.1371/journal.pone.0162708 (PMC5019492; doi:10.1371/journal.pone.0162708)
Supplement: S5 Table — List of primers used for qPCR and genotyping. All sequences are displayed in 5’ to 3’ orientation. (DOCX) [file pone.0162708.s008.docx]

**S3 Table. Sequences of primers used in this study.** List of primers used for qPCR, genotyping, and sequencing. All sequences are displayed in a 5’ to 3’ orientation.

| **qPCR primers** | | | |
| --- | --- | --- | --- |
| **Gene name (primer ID#)** | **WormBase Sequence Name** | **Forward primer sequence (5’-3’)** | **Reverse primer sequence (5’-3’)** |
| *fat-5* (SP313/14) | W06D12.3 | caactaccatcacaccttcc | cccgttcagtttcacagcc |
| *fat-6* (SP315/16) | VZK822L.1 | caacttccatcacacattccc | tcctcgttgaatatcacatcc |
| *fat-7* (SP317/18) | F10D2.9 | tttccaccacacattcccac | tcttcacttccgtgattggc |
| *acs-2* (SP305/06) | F28F8.2 | agtgagacttgacagttccg | cttgtaagagaggaatggctc |
| *cpt-5* (SP176/77) | F09F3.9 | cttccaaagtcagaagtggc | agaattgcttgatttcctccg |
| *sodh-1* (SP744/45) | K12G11.3 | attggttggaggacacgaag | gctcgtggcctttcttacag |
| *ddo-2* (SP2667/78) | F18E3.7 | gaagcgcagaagaggacatc | ttcaattgctgttccccaac |
| *dhs-18* (SP2669/70) | C45B11.3 | catccaaaactaccgggaac | tttactgctgcctcatcacg |
| *icl-1* (SP309/10) | C05E4.9 | tctccgtggtatccatgcc | tgatcgaaaactctcttagcc |
| *fmo-2* (SP711/12) | K08C7.5 | ggaacaagcgtgttgctgt | gccatagagaagaccatgtcg |
| K05B2.4 (SP699/700) | K05B2.4 | ccctatacgaatgacaggattg | tgtttgaaccttgtggtgag |
| Y40B10A.7 (SP679/80) | Y40B10A.7 | caccggagcatctgcattag | gcatttggagaggattggaac |
| *lips-6* (SP2575/76) | ZK617.2 | agaaggcttcggaattcacc | cttggaaagaccagcagtgg |
| *tag-38* (SP2646/47) | B0222.4 | ctggacaaccaatgatggag | actccagctgggaattgaag |
| Y65B4BR.1 (SP2571/72) | Y65B4BR.1 | cctccatgtgttcgagtgc | gaatctcctgctgagcatcc |
| *oac-56* (SP2577/78) | Y67A10A.1 | tacttcgctttgggatttgg | tggcgcaaatctacaataacc |
| W02B12.1 (SP2573/4) | W02B12.1 | atttttcaacgggattctgg | atatttccatgcccgtaagc |
| *act-1*(SP621/22) | T04C12.6 | gctggacgtgatcttactgattacc | gtagcagagcttctccttgatgtc |
| *tba-1* (SP1231/31) | F26E4.8 | gtacactccactgatctctgctgacaag | ctctgtacaagaggcaaacagccatg |
| *ubc-2* (SP1228/29) | M7.1 | agggaggtgtcttcttcctcac | cggatttggatcacagagcagc |
| **Genotyping and sequencing primers** | | | |
| **Gene name (primer ID#)**  **Genotype Strain** | **WormBase Sequence Name** | **Forward primer sequence (5’-3’)** | **Reverse primer sequence (5’-3’)** |
| *nhr-49* (SP2365/66)  *nhr-49(et7)* internal | K10C3.6 | aatgctctcagcaactcttgcagcgct | gtgattggagaggatgaattgccactg |
| *nhr-49* (SP2367/68)  *nhr-49(et7)* external | K10C3.6 | atctccgacaacgttaccgactaatggg | gggaaaagatgaaagatcacagattcgg |
| *nhr-49* (SP2369/70)  *nhr-49(et8)* internal | K10C3.6 | aatgatcattcgacggctccggtatt | tgtcggagatgaaagatgttgctgtaca |
| *nhr-49* (SP2371/72)  *nhr-49(et8)* external | K10C3.6 | acccagaagccaaaggactctcgaatag | ctgatccggctggatttaacggagatac |
| *nhr-49* (SP2373/74)  *nhr-49(et13)* internal | K10C3.6 | tgaatccgtacataaatgatagcttcaag | aatgaactcggagagcagagaattct |
| *nhr-49* (SP2375/76)  *nhr-49(et13)* external | K10C3.6 | ttgtatgtttgaaagcacttgcattctt | aattttacggtttttccctgaaatcatt |
| *nhr-49* (SP1764/65)  *nhr-49(nr2041)* external | K10C3.6 | ttccgccgaacagttcttcg | cgtcatctggcatctgatcg |
| *nhr-49* (SP1773/74)  *nhr-49(nr2041)* internal | K10C3.6 | tcatctgaatcacatccacg | cctggagatttcattcagatcc |
| *nhr-49* (SP2892)  sequencing | K10C3.6 | catcaatgatcattcgacggc |  |
| *nhr-49* (SP2913)  sequencing | K10C3.6 | tgttgttggcaccggctctc |  |
| *nhr-66* (SP2450/51) *nhr-66(ok940)* external | T09A12.4 | ctgcagcaagtcgaggttcg | gttgtctctaccagcatacc |
| *lips-6* (SP3249)  sequencing | ZK617.2 | gagtcggagctgaaacttgtga |  |
